# Mesenchymal Stem Cells from Mouse Hair Follicles Inhibit the Development of Type 1 Diabetes

**DOI:** 10.3390/ijms25115974

**Published:** 2024-05-29

**Authors:** Dragica Mićanović, Suzana Stanisavljević, Hanluo Li, Ivan Koprivica, Natalija Jonić, Ivana Stojanović, Vuk Savković, Tamara Saksida

**Affiliations:** 1 Department of Immunology, Institute for Biological Research “Siniša Stanković”, National Institute of Republic of Serbia, University of Belgrade, Bulevar Despota Stefana 142, 11060 Belgrade, Serbia; dragica.gajic@ibiss.bg.ac.rs (D.M.); ssuzana@ibiss.bg.ac.rs (S.S.); ivan.koprivica@ibiss.bg.ac.rs (I.K.); natalija.jonic@ibiss.bg.ac.rs (N.J.); ivana@ibiss.bg.ac.rs (I.S.); cvjetica@ibiss.bg.ac.rs (T.S.); 2 National “111” Center for Cellular Regulation and Molecular Pharmaceutics, Cooperative Innovation Center of Industrial Fermentation (Ministry of Education & Hubei Province), Hubei University of Technology, Wuhan 430068, China; lihanluo@hbut.edu.cn; 3 Department of Cranial Maxillofacial Plastic Surgery, University Clinic Leipzig, 04103 Leipzig, Germany

**Keywords:** MSC therapy, autoimmunity, immunomodulation, streptozotocin, mouse model, insulin expression

## Abstract

Mesenchymal stem cells (MSCs) are known for their immunosuppressive properties. Based on the demonstrated anti-inflammatory effect of mouse MSCs from hair follicles (moMSCORS) in a murine wound closure model, this study evaluates their potential for preventing type 1 diabetes (T1D) in C57BL/6 mice. T1D was induced in C57BL/6 mice by repeated low doses of streptozotocin. moMSCORS were injected intravenously on weekly basis. moMSCORS reduced T1D incidence, the insulitis stage, and preserved insulin production in treated animals. moMSCORS primarily exerted immunomodulatory effects by inhibiting CD4^+^ T cell proliferation and activation. Ex vivo analysis indicated that moMSCORS modified the cellular immune profile within pancreatic lymph nodes and pancreatic infiltrates by reducing the numbers of M1 pro-inflammatory macrophages and T helper 17 cells and upscaling the immunosuppressive T regulatory cells. The proportion of pathogenic insulin-specific CD4^+^ T cells was down-scaled in the lymph nodes, likely via soluble factors. The moMSCORS detected in the pancreatic infiltrates of treated mice presumably exerted the observed suppressive effect on CD4^+^ through direct contact. moMSCORS alleviated T1D symptoms in the mouse, qualifying as a candidate for therapeutic products by multiple advantages: non-invasive sampling by epilation, easy access, permanent availability, scalability, and benefits of auto-transplantation.

## 1. Introduction

Type 1 diabetes (T1D) is an autoimmune disease caused by the targeting of autoantigens derived from pancreatic β cells by one’s own immune cells and subsequent induction of their destruction. Since pancreatic β cells produce insulin, their obliteration leads to insulin deficiency and consequently to hyperglycemia [1]. The autoimmune process in T1D has a complex milieu that involves several types of immune cells. The CD8^+^ T cells are the most abundant population of immune cells in insulitis lesions during the pathogenesis of T1D, followed by macrophages, CD4^+^ T cells, B lymphocytes, and plasma cells [2], as well as rare regulatory T cells (Treg) and natural killer (NK) cells. Despite many studies that have investigated the development of T1D, the exact triggers of the autoimmune process have remained elusive.

The pancreatic anatomy and its morphological position in the body limit the prospects of investigating T1D in humans. The available peripheral blood samples and rare autopsies or biopsies of the pancreas do not provide relevant information. Therefore, the use of animal models is required for the development of new therapeutic approaches. Mouse models suitable for studying T1D include either non-obese diabetic (NOD) mice, which spontaneously develop T1D [3], or employ chemically induced T1D by intraperitoneal injections of streptozotocin (STZ) or alloxan [4].

Treatment of T1D has been increasingly addressed by stem cell-based therapies, in particular mesenchymal stem cells (MSCs). MSCs are multipotent cells capable of differentiating into various cell types, including mesodermal, ectodermal, or endodermal lineages [5]. This ability has sparked interest in MSC research, and the biology and therapeutic potential of MSCs have been well described. Due to overlaps of MSC qualities with other cell types, the International Society for Cellular Therapy (ISCT) has established minimal criteria for distinguishing MSCs. This minimal ISCT panel includes adherence to plastic, in vitro differentiation into osteoblasts, adipocytes, and chondroblasts, as well as high expression of the surface molecules CD105, CD73, and CD90 with simultaneous lack of expression of CD45, CD34, and CD14, or CD11b and CD79a, or CD19 and HLA class II [6]. Adult MSCs are most frequently isolated from bone marrow, adipose tissue, and muscle, but many other sources of MSCs have been identified, such as blood, retina, fallopian tubes, synovial fluid, amniotic fluid, umbilical cord, placenta endometrium, dental pulp, urine, and gingiva [7,8,9,10,11,12].

MSCs possess anti-inflammatory, anti-apoptotic, anti-tumorigenic, anti-fibrotic, neuroprotective, and chemo-attractive qualities and immense therapeutic potential for tissue repair and regeneration [13]. Accordingly, MSCs have been shown to be protective in treatments of autoimmune diseases [14], such as T1D, and have been abundantly assessed in animal models [15]. Their immunomodulatory properties have primarily been identified as the inhibition of pro-inflammatory immune cells and stimulation of Treg [15]. For example, human adipose-derived MSCs decreased the incidence of T1D in NOD mice by reducing the accumulation of T cells and macrophages in pancreatic islets, consequently increasing the insulin content and the size of the preserved islet beta cell area [16]. Bassi et al. [17] demonstrated an association of reversed hyperglycemia with the downregulation of T helper (Th) 1 and expansion of the Treg cell population in the pancreatic lymph node (PLN), along with reduced inflammatory cell infiltration and interferon-gamma (IFN-γ) levels in the pancreas. Moreover, not only cells but also their secreted content in the form of extracellular vesicles derived from human bone marrow MSCs prevented the onset of T1D, inhibited activation of antigen-presenting cells, and suppressed the development of Th1 and Th17 cells in the mixed lymphocyte reaction assay [18]. Human umbilical cord Wharton jelly-derived mesenchymal stem cells showed full and sustained remission of hyperglycemia throughout 216 days post-transfer in NOD mice with mild T1D [19].

Clearly, MSCs bring about great prospects for the treatment of T1D, as reflected by twenty-eight registered clinical trials, three of them being already completed by July 2023 (www.clinicaltrials.gov, accessed on 1 March 2024; searched by diabetes type 1 (condition or disease) and mesenchymal stem cells (intervention/treatment)).

Methods for the isolation of adult MSCs are usually invasive, involve surgical procedures, or yield only a small amount of tissue sufficient for processing and characterization, but not for upscaling to therapy-relevant quantities. The only known source of MSCs that is non-invasive, permanently available, and quickly upscalable is the outer root sheath of the hair follicle (ORS) [20,21]. The new MSC cell product isolated from mouse ORS (moMSCORS) showed the potential for differentiating into the chondrogenic, adipogenic, and osteogenic lineages, endothelial cells, and smooth muscle cells. Further, moMSCORS displayed a beneficial effect in the murine wound-healing model by means of their immunomodulatory properties [22], which provided a rationale for using them in an in vivo model of T1D. In this study, we tested the ability of moMSCORS to ameliorate T1D in an animal model of diabetes induced by multiple low doses (MLDS) of STZ. Specifically, we investigated the effects of moMSORS i.v. administration on histopathological changes and insulin expression in the pancreas of diabetic animals along with the frequency of immune cells relevant for T1D pathogenesis in the pancreatic lymph nodes and pancreas.

## 2. Results

### 2.1. moMSCORS Inhibit T Cell Proliferation and Activation In Vitro

**Figure 1:**
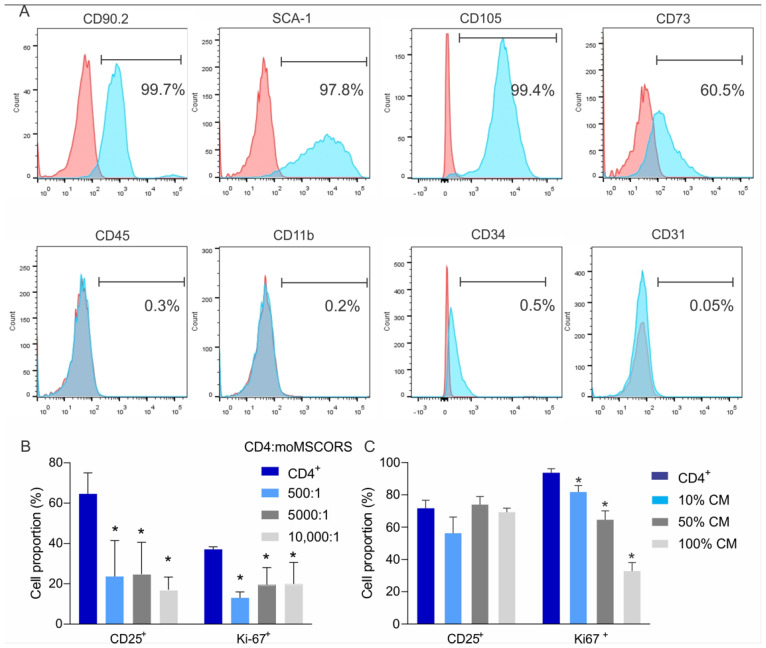
moMSCORS phenotype and co-cultivation with T cells. The expression of CD90.2, SCA-1, CD105, CD73, CD45, CD11b, CD34, and CD31 surface markers (blue line) and appropriate isotype control antibody (red line) on moMSORS (**A**). T cells were cultivated in different ratios with moMSCORS, and their activation (CD25^+^ cells) or proliferation (Ki67^+^ cells) was determined in direct contact with moMSCORS (**B**) or with moMSCORS-conditioned media (**C**). All measurements were performed on three samples per group, repeated twice. * *p* < 0.05 was considered as a statistically significant difference vs. CD4^+^ cells.

As previously described, moMSCORS were isolated from the whisker hair of the C57BL/6 mouse [23]. The identity of moMSCORS was verified according to their adherence to cell culture plastic, proliferation, colony forming, and the expression of CD90.1, SCA-1, CD105, and CD73, whereas the expression of negative markers CD45, CD11b, CD34, and CD31 was almost undetectable (Figure 1A).

To test the capacity of moMSCORS to inhibit T cell proliferation, we co-cultured purified CD4^+^ T lymphocytes with increasing numbers of moMSCORS for 72 h. In all ratios examined, the presence of moMSCORS coincided with a significant decrease in CD25 expression, hereby reaching a 3-fold difference in the 10,000:1 ratio (Figure 1B). Also, a reduced quota of Κi67^+^ cells, used as a criterion for proliferation, was observed (Figure 1B).

To test whether the observed inhibition was mediated via direct cell contact or via soluble markers, we cultivated T cells in the presence of supernatants obtained from moMSCORS cultivation. The moMSCORS-conditioned medium inhibited the proliferation of CD4^+^ cells in a dose-dependent manner, while the expression of CD25 remained unchanged (Figure 1C).

### 2.2. moMSCORS Ameliorates T1D in Mice

**Figure 2:**
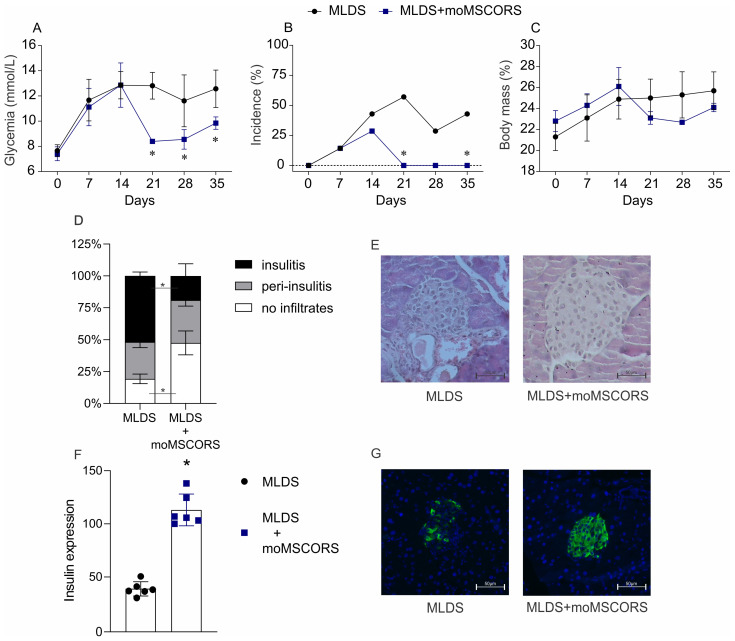
The effect of intravenous application of moMSCORS on T1D development in C57BL/6 mice. Glycemia levels (**A**), T1D incidence (**B**), body weight (**C**). Histological assessment of insulitis, i.e., the proportion of pancreatic islets with no immune cell infiltrates, surrounding infiltrates (peri-insulitis), or infiltrates within the islet (insulitis) (**D**). Representative histological sections (hematoxylin) (**E**). Quantification of insulin presence in the pancreatic sections(**F**). Representative histological sections (insulin is colored green) (**G**). All measurements were performed on samples from six animals per group. * *p* < 0.05 was considered as a statistically significant difference between MLDS and MLDS + moMSCORS.

MLDS-induced T1D in C57BL/6 mice served as the in vivo model for testing moMSCORS immunosuppressive properties. Evidently, i.v. application of moMSCORS successfully decreased hyperglycemia levels after three applications (Figure 2A). The beneficial effect of moMSCORS persisted until the end of the observation period (14 days after the last cell treatment). In addition, moMSCORS increased the number of asymptomatic mice as measured by T1D incidence on days 21 and 35 (none of the treated mice exhibited hyperglycemia) (Figure 2B), whereas their body weight change was not affected (Figure 2C). Interestingly, after the 21st day after T1D induction, none of the treated mice were hyperglycemic. At end of the experiment (day 35), histological evaluation of pancreatic islets revealed an increased proportion of healthy pancreatic islets and simultaneously a reduced number of islets with heavy immune cell infiltrations (insulitis) in the moMSCORS-treated group by 2.5-fold (Figure 2D). Figure 2E shows a representative image of an islet with insulitis in the control diabetic group of mice and one healthy islet from an moMSCORS-treated mouse. Concordantly with the histological assessment of insulitis, insulin expression was significantly preserved in the treated mice, hereby reaching a 2.5-fold difference (Figure 2F,G).

### 2.3. moMSCORS Treatment Decreases the Production of Pro-Inflammatory Cytokines

**Figure 3:**
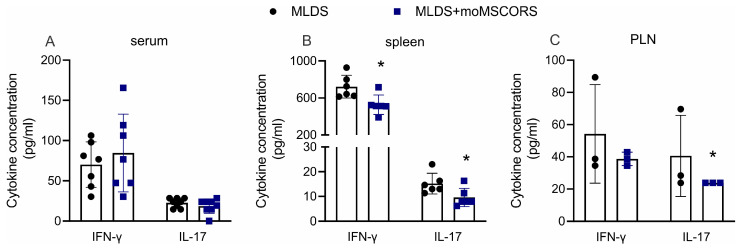
The effect of moMSCORS treatment on pro-inflammatory status during T1D. IFN-γ and IL-17 were quantified by ELISA in the serum (**A**), splenocyte supernatants (Con-A stimulated for IFN-γ or spontaneous for IL-17) (**B**), and in cultures of pancreatic lymph node cells (spontaneous secretion) (**C**). Representative results from one of two independent experiments performed are shown. * *p* < 0.05 was considered as a threshold for a statistically significant difference between MLDS and MLDS + moMSCORS.

To determine the effect of moMSCORS treatment on systemic and local immune responses, the concentration of pro-inflammatory cytokines was analyzed in the serum, spleen, and PLN cell culture supernatants. moMSCORS treatment did not affect the IFN-γ and IL-17 concentration in the serum of treated animals (Figure 3A), but it did significantly decrease their production in ConA-stimulated and non-stimulated splenocytes (for IFN-γ and IL-17, respectively) (Figure 3B).

The downregulatory effect of the moMSCORS treatment on spontaneous IL-17 secretion was evident only in vitro in the PLN cells (Figure 3C).

### 2.4. moMSCORS Treatment Affects the Proportion of Immune Cells in the Draining Lymph Node

**Figure 4:**
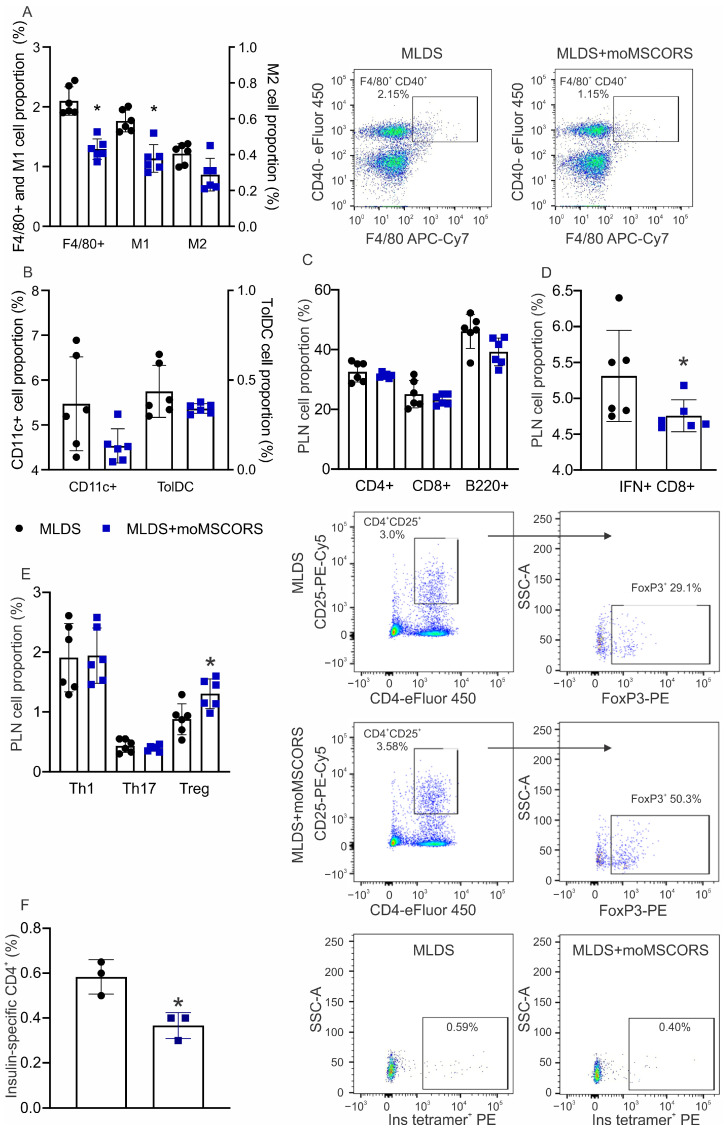
Ex vivo analysis of the immune cells within PLN. The proportions of macrophages (**A**), dendritic cells (**B**), T and B lymphocytes (**C**), IFN-γ^+^ CD8^+^ cytotoxic lymphocytes (**D**), the T helper cell profile (**E**), and insulin-specific CD4^+^ cells (**F**) were evaluated on day 10 after T1D induction by flow cytometry. Representative dot plots for cell types that were affected by the moMSCORS treatment are shown. * *p* < 0.05 was set as a threshold for statistical significance of the observed differences between MLDS and MLDS + moMSCORS.

Ex vivo analysis of myeloid cells in PLN revealed that moMSCORS treatment effectively decreased F4/80^+^ macrophages, particularly the pro-inflammatory M1 subset of macrophages (CD40^+^F4/80^+^ cells), whereas the anti-inflammatory M2 subset (CD206^+^F4/80^+^ cells) was not affected by the treatment (Figure 4A). No differences in total CD11c^+^ and tolerogenic dendritic cells (tolDC, CD11c^+^CD11b^−^CD103^+^) were observed between the moMSCORS-treated mice and the control group (Figure 4B). Analysis of the lymphocytic population showed no differences in CD4^+^, CD8^+^ T cell, and B cell proportions (Figure 4C). However, moMSCORS decreased the proportion of IFN-γ^+^ CD8^+^ cells (Figure 4D). Also, within the CD4^+^ T cells, the proportion of Treg was significantly increased, whereas the proportions of Th1 and Th17 remained the same as in MLDS-treated mice (Figure 4E). Importantly, the proportion of insulin-specific CD4^+^ T cells in PLN was significantly downregulated after the treatment with moMSCORS (Figure 4F).

### 2.5. moMSCORS Treatment Affects the Proportion of Immune Cells in the Pancreas

**Figure 5:**
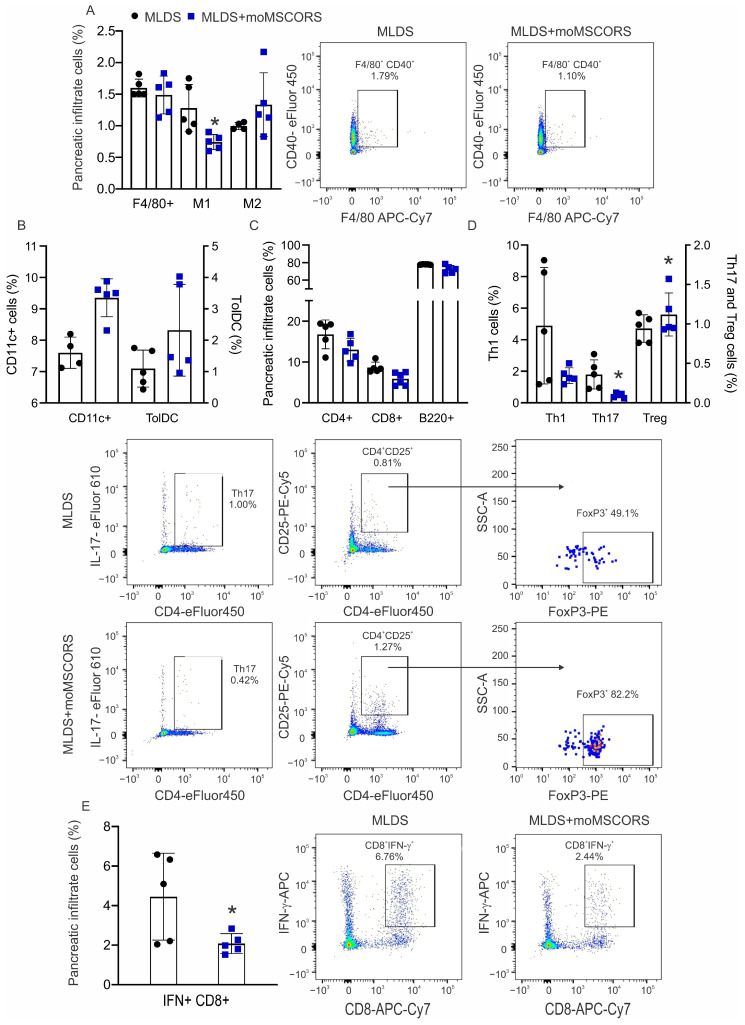
Ex vivo analysis of the immune cells within pancreatic infiltrates. The proportions of macrophages (**A**), dendritic cells (**B**), T and B lymphocytes (**C**), the T helper cell profile (**D**), IFN-γ^+^ CD8^+^ cytotoxic lymphocytes (**E**) were evaluated on day 10 after T1D induction by flow cytometry. Representative dot plots for cell types that were affected by the moMSCORS treatment are shown. Representative results from one of two independent experiments performed are shown. * *p* < 0.05 was considered as a statistically significant difference between MLDS and MLDS + moMSCORS.

Similarly to the effects observed in the PLN, moMSCORS treatment significantly decreased the proportion of M1 macrophages in the pancreatic infiltrates (Figure 5A), whilst no differences were observed in F4/80^+^ cells, M2 subset (Figure 5A), total CD11c^+^, and tolDC (Figure 5B). In terms of reflecting the proportions of lymphocytes in the PLN, there were no differences in CD4^+^, CD8^+^ T cells, and B cells between the control and moMSCORS-treated mice (Figure 5C). Also, Treg proportions were increased in the infiltrates of the treated mice (Figure 5D). Concomitantly with Treg upregulation, the Th17 population significantly decreased in the treated group, whereas no change was observed in the Th1 population between the groups (Figure 5D). Interestingly, the proportion of cells that can directly destroy pancreatic β cells during the autoimmune process, IFN-γ-producing CD8^+^ cytotoxic T lymphocytes, was significantly reduced in moMSCORS-treated mice (Figure 5E).

**Figure 6:**
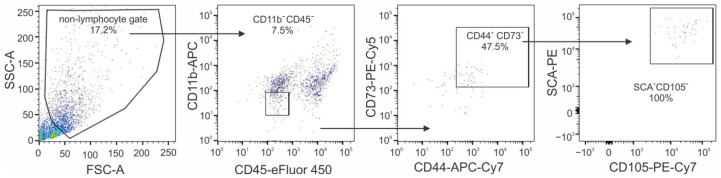
Ex vivo determination of moMSCORS’ presence in the pancreatic infiltrates. moMSCORS (CD45^−^CD11b^−^CD44^+^CD73^+^CD105^+^SCA-1^+^) were detected in the pancreatic infiltrates of the treated mice by flow cytometry, with the acquisition of 10,000 events in the first gate. Representative results from one of two independent experiments performed are shown.

Finally, the ability of moMSCORS to migrate to the pancreas of treated mice was investigated by flow cytometry. moMSCORS (CD45^−^CD11b^−^CD44^+^CD73^+^CD105^+^SCA-1^+^) were evident in the pancreatic cell suspension (around 4% of cells outside the lymphocyte gate), while at the same time, no signal indicating the presence of specific fluorescence was detected in the pancreas of MLDS-treated mice (Figure 6).

## 3. Discussion

This study demonstrates the protective effect of moMSCORS in the development of T1D in mice. The outcome showed that the intravenous administration of moMSCORS decreased the blood glucose level and infiltration of immune cells into the pancreas while preserving insulin expression. The observed effects of treatment by moMSCORS indicate that their effects are exerted by the downregulation of functional effector immune cells (Th17 and IFN-γ^+^ CD8^+^) and the stimulation of Tregs. Hereby, the main effect that can be ascribed to moMSCORS is immune modulation.

The moMSCORS have been reported as characterized according to the characterization panel for mesenchymal stem cells defined by the International Society for Cell Therapy, based on the paper of Dominici et al. [6]. The panel comprises adherence to the cell culture plastic, clonogenicity, proliferation, expression of phenotype-specific surface markers CD90.1, CD105, and CD73, as well as the capacity to differentiate into the adipogenic, osteogenic, and chondrogenic lineages. Beyond those minimal ISCT-defined requirements, the expression of surface markers CD44, Sca-1, CD140a, CD117, CD133, and CD11 in moMSCORS, along with their capacity to differentiate into endothelial and smooth muscle lineages has been routinely demonstrated [23]. The expression of nestin indicated their neuroectodermal identity rather than that of the primary mesenchyme. All of the abovementioned strongly implies that moMSCORS, along with their analogs human MSCORS [20,21] and equine eMSCORS [24], are true mesenchymal stem cells.

Nevertheless, we consider them a subset of the metapopulation of mesenchymal stromal cells, which encompass not only stem cells with diverse differentiating potential but also committed progenitors and differentiated cells [25]. The definitions and parameters for mesenchymal stem cells and mesenchymal stromal cells are progressively upgraded. The more accurate criteria can help in a precise delineation between the two on the nominal level, whereas in practice they can display largely overlapping phenotypes.

The umbilical cord and adipose tissue are the most common sources of human MSCs that have been explored for the therapy of various diseases, including T1D [15]. Although the umbilical cord, placenta, and Wharton’s jelly can be considered as “medical waste” and are easily accessible as a source of MSCs, our innovative candidate for a cell-based medicinal product is obtained even more easily from the hair follicle and in a completely non-invasive fashion. Additionally, the hair follicle and its outer root sheath remain available throughout most life phases, including senior age, which allows the scheduling of a sample at any time point. Importantly, the isolation of MSCs from hair follicles supports the possibility of autologous MSC transplantation, which, together with the aforementioned qualities, makes the hair follicle and the derived MSCs a versatile and highly personalized source of starting material for regenerative therapies. Although heterologous transplantation is not an issue with MSCs as they lack the expression of major histocompatibility complex II (MHC II) [26], the number of confounding variables (donor-to-donor variability, potential to initiate expression of MHC I and II) would surely be reduced in the case of using auto-transplantation with moMSCORS [22].

moMSCORS showed potent immunosuppressive properties in vitro and in vivo. The inhibition of T cell proliferation is in all probability related to a released soluble factor as the media conditioned by moMSCORS efficiently lowered the quantity of the Ki67^+^ CD4^+^ population. The possibility of moMSCORS producing IL-6 and vascular endothelial growth factor, which have already been demonstrated as being responsible for the anti-proliferative effect of bone marrow-derived mouse MSCs [27], needs to be focused on. Among the other possible mechanisms, the common denominator of several MSC preparations is the expression of indoleamine 2,3-dioxygenase (IDO) and subsequent production of prostaglandin E2 (PGE2) that inhibits T cells [28]. On the other hand, most of the mouse MSCs do not express IDO. In our results, there was no inhibition of T cell activation in the presence of moMSCORS’ media, suggesting that cell contact is mandatory for the activation blockade of T cells, represented by the expression of CD25. This particular finding is not in concordance with the reported claim of a damaging effect of MSCs on CD25^+^ cells, produced either by cleavage of CD25 from the surface of activated cells by the matrix metalloproteinase secreted by the MSCs or by an inhibition of CD25 expression in T cells by MSC-derived nitric oxide [29,30]. Although it creates a discrepancy in generally accepted available data, our finding indicates the possibility of a benevolent effect of direct contact between MScs and activated CD25+ cells, which remains to be further elucidated in future studies.

Administration of MSCs increased the level of insulin and reduced hyperglycemia in several studies employing animal models of T1D [31,32,33]. These data rationalize our study to test MSCs from an innovative source. As in these studies, moMSCORS showed a profound lowering effect on full insulitis development and consequently on disease incidence. The mechanism of action predominantly included a reduction in the frequency of M1 macrophages, Th17, and IFN^+^ CD8^+^ T lymphocytes, and the stimulation of Treg numbers both in the draining lymph nodes and within the pancreas. MSCs have indeed been shown to have an inhibitory effect on macrophages, which involves a multitude of biochemical cues and interacting cells. For example, genetically engineered MSCs inhibit M1 macrophages in vitro as well as after infusion to the liver in vivo [34]. MSCs were also shown to inhibit Th17 cell development through various ways, including inhibition of PGE2, IL-10, and PD-1/PD-L1 ligation [35,36]. Similar MSC-derived factors have been attributed to the MSC-mediated inhibition of activated CD8^+^ T cells [37]. This study’s results align with the literature’s data, such as the evident stimulatory effect of Wharton’s jelly-derived MSCs on splenic Treg in NOD mice [32]. Treatment of NOD mice with umbilical cord or bone marrow MSCs resulted in the same pattern that moMSCORS exerted, i.e., downregulation of Th17 and upregulation of Tregs in pancreatic lymph nodes [38]. On the other hand, Jurewicz et al. [32] did not observe any change in Treg expression but revealed an increase in TGF-β (Treg-stimulatory factor) in the pancreas.

One of the possibilities of beneficial actions of MSCs in T1D is their ability to differentiate into insulin-producing cells. MSCs derived from rat bone marrow can generate insulin-producing cells in vitro [39]. Also, MSCs from Wharton’s jelly differentiate into β cells in the presence of exendin-4 [40]. MSC-mediated stimulation of other pancreatic cell types to convert into β cells also presents a candidate mechanism of MSC paracrine influence [41,42,43]. This differentiation potential of moMSCORS could be tested in future studies, especially in light of the evident moMSCORS migration to the pancreas of diabetic animals (Figure 6). The mere presence of moMSCORS in the pancreas can indicate a direct immunosuppressive effect of these cells on the existing inflammatory milieu. Similarly as in our findings, after an i.v. application of MSCs in the Wistar rat diabetes model, MSCs were detected in the pancreatic sections of diabetic animals, thus confirming their ability to migrate from the circulation into the inflamed tissue [44]. Another plausible mechanism of moMSCORS’ protective action is through facilitating pancreas regeneration. Research has shown that mesenchymal stem cells produce insulin growth factor-1 (IGF-1) [45] that could enable the regeneration process [45]. Pancreatic expression of IGF-I prevented islet infiltration and β-cell death in a T1D model, thereby potentially regenerating and protecting β-cell mass in T1D [46].

Numerous clinical trials have been performed with MSCs derived from various origins for the benefit of individuals with T1D [47,48].

Several considerations regarding MSCs and their therapeutical use require caution prior to the progressive steps of clinical translation. Among them are inter-donor variability, ex vivo expansion, senescence, immunogenicity, and cryopreservation, which can all present setbacks in cell therapy, [49]. Those setbacks can particularly affect the immunomodulatory traits of applied cells. In light of those challenges, moMSCORS and MSCORS in general remain promising. In moMSCORS, the syngeneic experimental approach minimizes the inter-donor variability, which is present in MSCORS isolation routines. The cells appear insensitive to cryopreservation and multiple routines of thawing and re-cryopreserving. Also, the MSCORS [21] as well as moMSCORS displayed no morphological signs of senescence in the in vitro culture. Nevertheless, the in vitro stability, in particular the senescence of MSCs, is often overlooked, and it warrants thorough examination, which should be attended to as a subject of future studies as well as being included in the pre-clinical development.

The latest published clinical trial performed in Sweden was a combined Phase I/II clinical trial in which treatment with allogeneic Wharton’s jelly MSCs was compared with a placebo in adults with newly diagnosed T1D. The results of that study suggest that the treatment is safe and has prospects for T1D treatment as insulin requirements in MSC-treated individuals did not change over the 12-month follow-up period, whereas insulin needs increased in placebo-treated individuals. As many studies (including the present one) indicate that a conditioned medium from MSCs could be a source of immunosuppressive effects [50], the use of a conditioned medium or exosomes instead of cells may be the future for MSC-based therapy in general.

## 4. Materials and Methods

### 4.1. Isolation of Mesenchymal Stem Cells from Mouse Whisker Hair Follicle Outer Root Sheath (moMSCORS)

The method to obtain moMSCORS from mouse whisker hair follicles was described in detail in our previous research [23]. Briefly, the excised facial skin area was disinfected and rinsed with PBS. The attached muscle, subdermal fat, and blood vessels were removed by maceration. Clean whisker pads were placed in 5 mL of collagenase type V (2 mg/mL; Sigma Aldrich, Heidelberg, Germany) and incubated for 3 h at 37 °C. After the collagenase digestion, the whisker hair shaft was clipped by a pair of forceps from the distal side and pushed in the proximal direction, opposite the hair growth. Extracted follicles with an intact outer root sheath were placed in a petri dish containing moMSCORS cell culture medium (DMEM low glucose + 10% fetal calf serum (FCS) + 2 mM L-glutamine + penicillin/streptomycin (100 U/mL/100 μg/mL)). The base follicle part with dermal papilla was cut off, and the shortened follicles were seeded onto 6-well-format Transwell meshes (Corning Inc., New York, NY, USA) to set up the air–liquid interface. The lower portion of the setup was filled with 0.9 mL/well of moMSCORS cell culture medium, which provided contact with the mesh and maintained a liquid film at the upper side of the follicles. Hair follicles were incubated under hypoxic conditions (5% O_2_, 5% CO_2_) at 37 °C, and the medium was changed every 2–3 days in the volume of 1.2 mL. Within the next 17–24 days, the cells left the ORS of the follicle and populated the mesh. After having reached 50–80% confluence, the cells were detached from the mesh using 0.04%/0.03% trypsin/EDTA, subcultured in 6-well plates and subsequently in T25 culture flasks under hypoxic conditions. In our previous routine [23], moMSCORS successfully differentiated into osteo-, chondro-, and adipogenic lineages under relevant conditions. Therefore, we standardized their characterization and reduced it to the expression of surface markers CD90.1, SCA-1, CD105, CD73, CD45, CD11b, CD34, and CD31. For in vivo studies, the cells were trypsinized and collected in passages 4 and 5 to be freshly applied to the tail vein (i.v.).

### 4.2. T1D Induction and moMSCORS Treatment

C57BL/6 mice were kept at the Animal Facility at the Institute for Biological Research “Siniša Stanković”, National Institute of Republic of Serbia, University of Belgrade, Serbia, under standard conditions with free access to food and tap water, with the addition of hiding structures for environmental enrichment. All experimental procedures were approved by the Veterinary Administration, Ministry of Agriculture, Forestry and Water Management, Republic of Serbia (No 323–07 09476/2021–05) and performed in accordance with Directive 2010/63/EU.

T1D was induced in male 6–8 week old C57BL/6 mice (22–25 g) by administration of MLDS. STZ (40 mg/kg bw; Sigma-Aldrich, St. Louis, MO, USA) was dissolved in cold 0.1 M citrate buffer (pH 6) prior to administration and given intraperitoneally for 5 consecutive days. The STZ-treated animals were divided into two groups (n = 10): the test group being injected with cells and the control group receiving PBS. The cell treatment was performed at the following time points: 3 h after the first STZ (day 0) and then again on days 7, 14, and 21. The animals were anesthetized by isoflurane and 5 × 10^5^ moMSCORS in 200 µL PBS were injected via the tail vein. The control MLDS-treated group received PBS in an equal volume. For disease monitoring and histological evaluation of the pancreas, the animals were sacrificed on the 35th day. Mice were monitored weekly for variations in body weight and for blood glycemia by means of a glucometer (GlucoSure, Apex Biotechnology Corp., Hsinchu, Taiwan). Hyperglycemia was defined as a blood glucose level higher than 11 mmol/l in non-fasted animals on three consecutive days, as described previously [51,52]. For the ex vivo analyses that were performed on day 10 after the first STZ, the moMSCORS were administered on day 0 and day 7.

### 4.3. Histological Analysis

On the 35th day post-STZ injection, pancreata were collected, paraffin-embedded, sectioned into 5 μm slices with 200 μm intervals, stained with Mayer’s hematoxylin (Bio-Optica, Milan, Italy), and examined using a Zeiss Imager Z1 microscope (Carl Zeiss Meditec AG, Oberkochen, Germany). Blinded insulitis scoring of at least 20 islets per pancreas was performed as follows: grade 0 was assigned to intact islets, grade 1 was assigned to peri-islet infiltrates, and grade 2 was assigned to heavy intra-islets infiltrates or destroyed islets, as previously described [52]. The results were expressed as the ratio of graded islets to the total number of examined islets.

To detect insulin, sections were stained with FITC-conjugated rabbit anti-mouse insulin antibody (1:400, Cell Signaling Technology, Boston, MA, USA) and counterstained with Hoechst 33342 dye (2 μL/mL, Invitrogen, Eugene, OR, USA). An appropriate negative control was used—the rabbit anti-goat IgG-biotin (Vector Laboratories, Newark, CA, USA) with streptavidin-FITC (ThermoFisher Scientific, Waltham, MA, USA). Images were acquired at 20× magnification using an AxioVision microscope (Carl Zeiss Meditec AG, Oberkochen, Germany). Insulin presence in islets was analyzed using Image J 1.46r software [53]. Images were converted to grayscale, and fluorescence intensity was quantified by measuring the mean gray value, representing the sum of gray values of all pixels in the selection divided by the total number of pixels.

### 4.4. Cell Isolation

Cells from the spleen and PLN were isolated by passing tissues through a 40 μm cell strainer and removing erythrocytes with RBC lysis buffer (ThermoFisher Scientific, Waltham, MA, USA). For obtaining the pancreatic infiltrates, the collagenase digestion method was employed. The pancreata were minced into small pieces and shaken in collagenase type V (Sigma-Aldrich, Heidelberg, Germany) solution for 15 min at 37 °C. The digested tissue was vortexed, overlaid on density gradient solution (HistoPaque-1083), and centrifuged at 2000 rpm for 20 min. Cells from the interface were collected, washed, and resuspended in culture medium (Table 1).

CD4+ cells were separated from the spleen cell suspension by incubation with biotin-conjugated anti-mouse CD4 antibody (1:60, eBioscience) and resuspension in magnetic bead buffer (Table 1.) with BD IMag™ Streptavidin Particles Plus–DM (1:20, BD Biosciences, San Hose, CA, USA). Cells were purified using a BD IMag™ Cell Separation Magnet (BD Biosciences, San Hose, CA, USA) for 3 × 8 min and resuspended in a T lymphocyte medium (Table 1).

### 4.5. In Vitro Suppression Assay

To study the suppressive activity of moMSCORS on CD4^+^ lymphocytes, CD4^+^ cells isolated from the spleen of healthy C57BL/6 mice were co-cultured with moMSCORS in different ratios (500:1, 5000:1, and 10,000:1). moMSCORS were seeded in a 24-well plate in the moMSCORS medium and incubated under hypoxic conditions overnight. Then, the wells were coated with anti-mouse CD3 antibody (1 μg/mL, eBioscience), and an equal number of purified CD4^+^ cells (1 × 10^6^) were placed in each well, and resuspended in the T lymphocyte medium. The cells were also stimulated by the addition of anti-mouse CD28 antibody (1 μg/mL, eBioscience, Frankfurt, Germany). After 72 h of cultivation, the cells were washed, stained as described below, and analyzed by flow cytometry. To test the suppressive activity of the moMSCORS-conditioned medium on CD4^+^ lymphocytes, purified CD4^+^ cells (1 × 10^6^) were seeded in a 24-well plate and incubated in the presence of a 10%, 50%, or 100% moMSCORS-conditioned medium. After 72 h of cultivation, the cells were washed, stained as described below, and analyzed by flow cytometry. The moMSCORS-conditioned medium was collected from the moMSCORS cell culture 48 h after the 5th passage.

### 4.6. Detection of Extracellular and Intracellular Markers Using Flow Cytometry

Surface molecules were detected on viable cells in suspension with PBS + 1% BSA. The used anti-mouse antibodies are listed in Table 2. Staining was performed for 45 min at 4 °C. Tregs were detected by the Mouse Regulatory T cell Staining Kit (FoxP3) according to the manufacturer’s instructions (eBioscience, Frankfurt, Germany).

For intracellular cytokine staining, cells were stimulated with a cell stimulation cocktail (plus protein transport inhibitors) (1:500, eBioscience, Frankfurt, Germany) for 4 h at 37 °C. Cells were fixed in 2% paraformaldehyde, permeabilized, and stained with the antibodies listed in Table 3. Appropriate isotype-matched controls (eBioscience) were included in all experiments. For the Ki67-FITC (goat polyclonal) antibody (Santa Cruz Biotechnology, Heidelberg, Germany), cells were permeabilized as for FoxP3/Treg detection. The acquisition was undertaken using Partec CyFlow Space (Partec, Görlitz, Germany) and analyzed on FlowMax software version 2. 82 or on FACSCalibur and BD FACSAria III (BD Biosciences), and analyzed with FlowJo VX software version 10 (Treestar, Ashland, OR, USA). Cells were first gated on live cells and then further gated as needed for analysis.

### 4.7. ELISA

Splenocytes were cultured in the presence of Concanavalin A (ConA, 1 μg/mL) for 72 h. the cytokine concentration in cell culture supernatants was determined by sandwich ELISA, which was performed in MaxiSorp plates (Nunck, Rochild, Denmark). PLN cells were cultured without stimulation for 72 h. Anti-mouse IFN-γ and IL-17 (R&D Systems, Minneapolis, MN, USA) paired antibodies were used according to the manufacturer’s instructions. Absorbance was measured by the Synergy H1M Multi-Mode Microplate Reader (BioTek, Winooski, VT, USA) at 450 and 570 nm. A standard curve created from the known concentrations of appropriate recombinant cytokines was used to calculate the concentration values of measured cytokines.

### 4.8. Statistical Analysis

Data are presented as mean ± SD. The results for T1D monitoring are presented as a representative of four repeated experiments, while ex vivo analyses are presented as a representative of two repeated experiments with similar results. The significance of differences between groups was determined by a two-tailed Student’s *t*-test or Mann–Whitney non-parametric test. The threshold for statistical significance of observed differences was set at *p* < 0.05. Statistical analyses were performed using GraphPad Prism 8 software (GraphPad Software, Inc., La Jolla, CA, USA).

## 5. Conclusions

In conclusion, to our knowledge, the present study is the first research to demonstrate the in vivo effect of MSCs isolated from hair follicles on T1D induction and development and to determine their immunosuppressive activity as well as identify the anti-inflammatory mediation by Treg enhancement and through the inhibition of pathogenic T cells.

## Figures and Tables

**Table 1:** Solution compositions used for cell isolation.

Medium for cell culture	RPMI 1640 1% Pen/Strep 2 mM L-glutamine 25 mM HEPES 5% FCS
Magnetic bead buffer	PBS 0.5% BSA 2.5 mM EDTA
T lymphocyte medium	RPMI 1640 1% Pen/Strep 0.02 mM Na-pyruvate 5 μM β-mercaptoethanol 2 mM L-glutamine 25 mM HEPES 10% FCS

**Table 2:** Anti-mouse antibodies specific for surface molecules used in flow cytometry.

Antibody	Fluorophore	Host	Manufacturer
CD45	eFluor™506	rat IgG2b, κ	Invitrogen, Waltham, MA, USA
CD4	eFluor™450	rat IgG2a, κ	
CD8	APC-eFluor™780	rat IgG2a, κ	
CD25	PE	rat IgG1, λ	
CD25	PE-Cy5.5	rat IgG1, λ	
B220	Alexa Fluor™700	rat IgG2a, κ	
F4/80	APC-eFluor™780	rat IgG2a, κ	
CD40	eFluor™450	Armenian hamster IgM, κ	
CD206	eFluor™660	rat IgG2b, κ	
CD11b	PE	rat IgG2b, κ	
CD11b	APC	rat IgG2b, κ	
CD11c	PE-Cy5	Armenian hamster IgG	
CD90.2	FITC	rat IgG2a, κ	
CD73	PerCP-eFluor™710	rat IgG1	
CD44	APC-eFluor™780	rat IgG2b, κ	
CD103	PE/Dazzle™594	Armenian hamster IgG	BioLegend, San Diego, CA, USA
CD105	PE-Cy7	rat IgG2a, κ	
Sca-1	PE	rat IgG2a, κ	

**Table 3:** Anti-mouse antibodies specific for intracellular molecules used in flow cytometry.

Antibody	Fluorophore	Host	Manufacturer
IFN-γ	APC	rat IgG1, κ	Invitrogen, Waltham, MA, USA
IL-17	eFluor™610	rat IgG1, κ	
IL-10	FITC	rat IgG2b, κ	

## Data Availability

The data presented in this study are available on request from the corresponding author.
